# Hepatitis Delta Virus Detected in Salivary Glands of Sjögren's Syndrome Patients and Recapitulates a Sjögren's Syndrome-Like Phenotype *in Vivo*

**DOI:** 10.20411/pai.v1i1.72

**Published:** 2016-05-23

**Authors:** Melodie L. Weller, Matthew R. Gardener, Zoe C. Bogus, Michael A. Smith, Elisa Astorri, Drew G. Michael, Donald A. Michael, Changyu Zheng, Peter D. Burbelo, Zhennan Lai, Paul A. Wilson, William Swaim, Beverly Handelman, Sandra A. Afione, Michele Bombardieri, John A. Chiorini

**Affiliations:** 1 Molecular Physiology and Therapeutics Branch, National Institute of Dental and Craniofacial Research, National Institutes of Health, Bethesda, Maryland; 2 Centre for Experimental Medicine & Rheumatology, William Harvey Research Institute, Barts and The London School of Medicine & Dentistry, Queen Mary University of London, London, UK; 3 Dental Clinical Research Core, National Institute of Dental and Craniofacial Research, National Institutes of Health, Bethesda, Maryland; 4 National Intramural Database, Division of Enterprise and Custom Applications, Center for Information Technology, National Institutes of Health, Bethesda, Maryland

**Keywords:** Sjogren's syndrome, Sjögren's syndrome, Viral-mediated autoimmunity, Hepatitis Delta Virus, Hepatitis D Virus, HDV, xerostomia, xerophthalmia, Sjogrens syndrome, Sjögrens syndrome

## Abstract

**Background:**

Low-level, chronic viral infections have been suspect in the development of select autoimmune diseases, including primary Sjögren's syndrome (pSS). Multiple studies have shown stimulation of antiviral response pathways in pSS tissues suggestive of a viral infection. Yet, with this data in hand, a causal link between a viral infection and development of pSS had not been identified. Therefore, a study was designed to further define the viral landscape within pSS-affected salivary gland tissue to identify potential viral-mediated triggers in the pathogenesis of this autoimmune disease.

**Methods:**

A viral microarray was utilized to measure viral transcripts present in salivary gland tissue from patients diagnosed with pSS compared to healthy controls. Murine models of salivary gland localized HDV antigen expression were developed to evaluate the capacity of a chronic HDV signature to trigger the development of a pSS-like phenotype.

**Results:**

Through this analysis, two distinct viral profiles were identified, including the increased presence of hepatitis delta virus (HDV) in 50% of pSS patients evaluated. Presence of HDV antigen and sequence were confirmed in minor salivary gland tissue. Patients with elevated HDV levels in salivary gland tissue were negative for detectible hepatitis B virus (HBV) surface antigen and antibodies to HBV or HDV. Expression of HDV antigens *in vivo* resulted in reduced stimulated saliva flow, increase in focal lymphocytic infiltrates, and development of autoantibodies.

**Conclusion:**

Identification of HDV in pSS patients and induction of a complete pSS-like phenotype *in vivo* provides further support of a viral-mediated etiopathology in the development of pSS.

**STANDFIRST**

Hepatitis delta virus has been detected in a subset of Sjögren's syndrome patients and shows capacity to trigger a similar disease phenotype *in vivo*.

## INTRODUCTION

Viral infections are thought to play a role in the development of chronic diseases, including autoimmune diseases [1]. While evidence of an antiviral response has been observed in multiple autoimmune diseases [2–8], association with a viral agent has not always been clear. This may be attributed to a multifactorial etiology of chronic autoimmunity requiring a genetic susceptibility component in combination with a persistent viral infection or chronic viral exposure to trigger disease pathogenesis. Therefore, a study was designed to further define the viral landscape and potential viral triggers of autoimmunity in disease-affected tissue as illustrated in patients with primary Sjögren's syndrome.

Primary Sjögren's syndrome (pSS) is an autoimmune disease estimated to affect over 35 million people worldwide [9, 10]. This autoimmune disease is currently diagnosed by a reduction in tear and/or saliva secretion, accumulation of focal lymphocytic infiltrates in salivary gland tissue, and development of antibodies against Ro(SSA), La(SSB), and nuclear proteins [11]. Epidemiological studies have reported patients experiencing subjective xerostomia and development of autoantibodies over a decade prior to diagnosis, suggesting a chronic, slowly progressing development of the disease pathology [12–14]. While the underlying trigger(s) of Sjögren's syndrome remains to be defined, various studies have pointed to an altered genetic susceptibility in connection with environmental exposures, including viral infections.

A common antiviral signature in combination with divergent symptomology suggests a multivariate etiology underlying the Sjögren's syndrome phenotype. Clinical studies have reported unique groups within the pSS patient population presenting with different symptom profiles and disease progression based on age of onset, sex, immunological presentation, and extraglandular involvement across a large cohort of pSS patients [15]. Gene expression analyses within affected salivary gland tissue have identified stimulation of antiviral response pathways, including upregulation of type I interferon (IFN) inducible genes and upstream viral-sensing Toll-like receptors [8, 16–18]. Although these studies echo a similar overlying antiviral response, the divergent clinical characteristics across the pSS patient population may be due to multiple etiologies, and potentially different viral infections, behind the collective phenotypic presentation of primary Sjögren's syndrome. Prior studies have detected the presence of viruses in salivary glands of Sjögren's syndrome patients, including Epstein-Barr Virus (EBV), Coxsackie virus, and Human T-lymphotropic virus (HTLV-1) [19–22]. Yet, even with the discovery of these viruses in Sjögren's syndrome patients, a clear connection between these viral infections and the development of primary Sjögren's syndrome has been difficult to establish.

To globally identify potential viral triggers in the pathogenesis of Sjögren's syndrome, a custom viral microarray was designed to identify low-level transcripts from actively replicating viruses present within salivary gland tissue of primary Sjögren's syndrome patients compared to healthy controls. Through this analysis, multiple virus profiles were identified in the pSS patient cohort evaluated. One of the virus profiles identified included the presence of hepatitis delta virus (HDV). Taking the next step, we evaluated the capacity of HDV antigens to trigger a Sjögren's syndrome-like phenotype *in vivo*.

## MATERIALS AND METHODS

### Patients and Samples

All patient studies were reviewed and approved by the Institutional Review Board USA (NCT00001390) and all patients signed a written consent form prior to enrollment. All primary Sjögren's syndrome patients met American-European Consensus Group (AECG) criteria [11] for diagnosis of Sjögren's syndrome. Clinical parameters and serum and salivary gland labial biopsies from 14 healthy controls and 15 patients diagnosed with primary Sjögren's syndrome were utilized in this study and are summarized in Supplemental Table 1.

Patients from the second United Kingdom (UK) cohort was approved by the hospital Ethics Committee (REC 05/Q0702/1) and all patients signed a written informed consent. This second dataset, summarized in Supplemental Table 1, included RNA samples isolated from labial salivary gland biopsies obtained from a total of 12 pSS patients and minor salivary gland biopsies from 10 sicca patients. Sicca samples were defined as patients experiencing select characteristics of pSS but did not meet full criteria for diagnosis of Sjögren's syndrome at time of tissue acquisition. An additional confirmation study, consisting of 8 pSS and 8 sicca samples, including samples (two positive and two negative) overlapping with those tested in the second phase of the study conducted at the NIH, were analyzed for independent confirmation of HDV in RNA isolated from minor salivary gland biopsies conducted at the UK location.

### Microarray

Viral microarray probes were designed using a modified method reported by Wang *et al*. [23]. Briefly, viral genomes were fragmented into 70nt sequences and iterated across 15nt offsets to produce up to 30,000 candidate probes per viral sequence. The resulting 70mer fragments were then blasted against the RefSeq nucleotide database, and probes with *E*-values < 0.05 were retained. These 70mer sequences were further filtered using Agilent eArray parameters for efficient melting temperature and nucleotide composition rendering optimized 60mer viral microarray probe sequences. A maximum of 5 probes per viral sequence was retained. Each array contained over 3000 probes that recognized putative conserved regions in viruses known to infect animals and covered 31 viral families. RNA isolated from minor salivary gland tissue was processed as previously reported [24].

Genespring (v.12.0) software was utilized for viral microarray analysis. Microarray data were normalized using quantile normalization and filtered for background intensity (threshold set to 25% of any one group having a raw probe intensity of > 100). Dataset evaluated in this study have been deposited in NCBI GEO Database (series no. GSE77599). Collective analysis identified probes significantly increased across the collective pSS patient group compared to the collective healthy controls and that presented a fold change > 2.0 in pSS relative to healthy controls. Statistical significance was corrected for multiple comparisons using the Benjamini-Hochberg correction method. Subgroup analysis was performed through identifying normalized the probe intensity of each probe and identifying probes for each individual patient that had a greater than a 95% confidence interval of the probe intensity for the collective healthy controls. Probes selected for further analysis presented with intensities greater than the 95% confidence interval of healthy controls and were present in at least 25% of the pSS patient samples tested. Pairwise correlations between normalized probe intensities of probes present in subgroups of pSS were then identified and mapped using JMP (v.10.0) statistical software from SAS. Heatmap and cluster analysis were generated using CIMminer (NCI).

### PCR Detection of HDV Sequence

Reverse transcription of RNA isolated from minor salivary gland biopsies was performed using Life Technologies Superscript II and random hexamer primers as per the manufacturer's specifications. The genomic HDV sequence was identified in RNA using a modified nested PCR classically used in the detection of HDV in HBV:HDV co-infections [25]. Modification to protocol included the addition of dNTPs to the first round of nested PCR. The transcript targeted nested RT-qPCR design used to detect both HDV genomic and transcript sequences and, in our testing, was more sensitive than the Smedile *et al*. PCR used to detect only the HDV genome.

The first round PCR was performed using Qiagen HotStarTaq DNA Polymerase as follows: 5μl of Q-solution; 3.5μl of 25 mM MgCl2; 2.5μl of 10X PCR buffer; 0.25μl Hot Start enzyme; 0.25μl 10mM dNTPs; 0.125μl 100mM tHDV-1F (5′- GGCTACTCTTCTTTCCCTTCTC-3′) and 0.125μl 100mM tHDV-1R (5′- ACAAGGAGAGGCAGGATCA-3′); 1μl cDNA; and PCR grade water to 25μl total volume. First round PCR conditions: 1 cycle of 5:00 minutes at 95°C; 35 cycles of 0:30 minutes at 94°C; 0:30 minutes at 50°C; 0:30 minutes at 72°C; and 1 cycle of 10:00 minutes at 72°C. The second round of qPCR was performed using the following primer and probe set: tHDV-2F (5′- TCTCGTCTTCCTCGGTCAA-3′); tHDV-2R (5′-GCCCTCGAGAACAAGAAGAA -3′); and tHDV-probe (5′-FAM/TTCCTCCTTGCTGAGGTTCTTGCC/3′-TAM-Sp). The qPCR reaction was performed on an Applied Biosystems ABI 7700 instrument and the reaction contained Applied Biosystems (ABI) TaqMan 2X PCR master mix, 500nM of primers, 250nM of probe, 1-3μl of first round PCR reaction, and PCR grade water to a total volume of 25μl. Increasing the volume of first-round PCR added to the nested reaction may be required to quantify lower levels of HDV sequence present in samples. The qPCR reaction was performed as follows: 1 cycle at 50°C for 2:00 minutes; 1 cycle at 95°C for 10:00 minutes; 40 cycles of 0:15 minutes at 95°C, 0:30 minutes at 50°C, and 1:00 minutes at 60°C. Quantitation of GAPDH was performed using Applied Biosystems (ABI) FAM/MGB labeled probe to human GAPDH (catalog # HS99999905_m1) and an assay was performed as outlined in the manufacturer's suggested protocol. HDV levels were calculated as fold change in pSS relative to average of healthy controls as per ABI protocol [26]. A more detailed protocol is available upon request.

### Detection of HDAg Expression in Salivary Gland Tissue

Immunohistochemical analysis of HDV antigens (HDAg) was performed using rabbit anti-HDAg antiserum as previously characterized [27, 28] for human salivary gland tissues. Staining of formalin fixed paraffin embedded (FFPE) human salivary gland tissue was performed using citrate (10mM sodium citrate, pH6.0, 0.05% Tween20) antigen retrieval, followed by 30 minute incubation in blocking solution (3% BSA in TRIS buffered saline [TBS]) at room temperature, incubation with primary antibody overnight at 4°C (1:400 for rabbit antiserum) and 1 hour room temperature incubation in labeled secondary antibody solution (Alexa Fluor 488 Goat Anti-Rabbit IgG, 1:500, Life Technologies). Slides were then mounted in Fluoromount G (Electron Microscopy Systems) containing DAPI counterstain. B-cell, (B220, Abcam catalog #AB64100, 1:250) and T-cells (CD3, Abcam, catalog #AB16669, 1:100) were detected in FFPE mouse tissues using the manufacturer's recommended conditions. Secondary species-specific antibodies for B-cell b220 (Alexa Fluor 594 Goat Anti-Rat IgG, 1:500, Life Technologies) and T-cell CD3 (Alexa Fluor 488 Goat Anti-Rabbit IgG, 1:500, Life Technologies) were incubated for 1 hour at room temperature and mounted in Fluoromount G (Electron Microscopy Systems) containing DAPI counterstain.

### Detection of Antibody Profiles by ELISA

ELISAs from International Immunodiagnostics (Foster City, CA) to detect antibodies to HDAg (catalog #277) and Abnova Corporation to detect antibodies to HBV core protein (catalog #KA0288) were performed on serum from healthy controls and pSS patients as per manufacturer's suggested protocols. A luciferase immunoprecipitation system (LIPS) was performed to detect HBV core protein or HDV antigen tagged with *Renilla* luciferase and was performed as previously published [29, 30]. A human anti-HDAg antibody was used as the positive control (gift from John Casey, PhD, Georgetown University).

Anti-SSA/Ro, anti-SSB/La, and antinuclear antibodies (ANA) were detected by ELISA from Alpha Diagnostics International using human serum as per the manufacturer's protocol. Total IgG (eBiosences), anti-SSA/Ro (Alpha Diagnostics International), anti-SSB/La (Alpha Diagnostics International), and ANA (Alpha Diagnostics International) were detected in mouse serum by ELISA as per the manufacturer's suggested protocols.

### Animal Model

All animal studies were approved by the NIDCR Institutional Animal Care and Use Committee (IACUC) and performed in compliance with the NIH Guide for the Care and Use of Laboratory Animals. Recombinant adeno-associated virus serotype 2 (AAV2) was produced and utilized for cannulation of submandibular salivary glands in 8-week-old female C57BL/6 mice as previously reported [31]. Mice were cannulated with 1.0x10^10^ genomic particle/gland AAV2 containing S-HDAg or L-HDAg sequences and spiked with AAV containing luciferase transgene as a control for cannulation efficacy. The combined expression of S-HDAg and L-HDAg (S-HDAg/L-HDAg) was facilitated by delivery of a 1:1 mixture of AAV containing S-HDAg or L-HDAg. Control mice were cannulated with AAV containing luciferase transgene. Viral aliquots of rAAV2-HDAg used for cannulation were spiked with 10% rAAV2-luciferase to confirm effective cannulation. One week post-cannulation, mice were monitored for luciferase expression in the salivary gland tissue region as previously reported [32]. Mice that had detectible levels of luciferase activity were utilized for the study and were assessed for pilocarpine stimulated saliva flow, antibody development, lymphocytic foci development and HDAg expression at 4 months post-cannulation using the methodology previously reported [24].

## RESULTS

Viral microarray analysis was performed using RNA isolated from minor salivary gland tissue from 15 primary Sjögren's syndrome patients and 14 healthy controls (Supplemental Table 1). The viral microarray contained over 3000 probes for viral families known to infect animals. Probes were designed to detect homologous sequences shared between multiple viral family members, enabling the detection of viral signatures with a limited number of probes [23, 33]. This method has the potential to identify transcripts of actively replicating RNA and DNA viruses within the affected salivary gland tissue. Our hypothesis was that a viral-mediated pSS-like phenotype may be caused by more than one type of viral infection. Therefore, the analysis of the viral array data was performed using two different approaches: (1) identification of the collective pSS patient cohort viral signature compared to the healthy controls; and (2) identification of individual viral signatures to identify subgroups within the pSS patient cohort compared to healthy controls.

### Identified Viral Profiles in pSS

**Figure 1.**
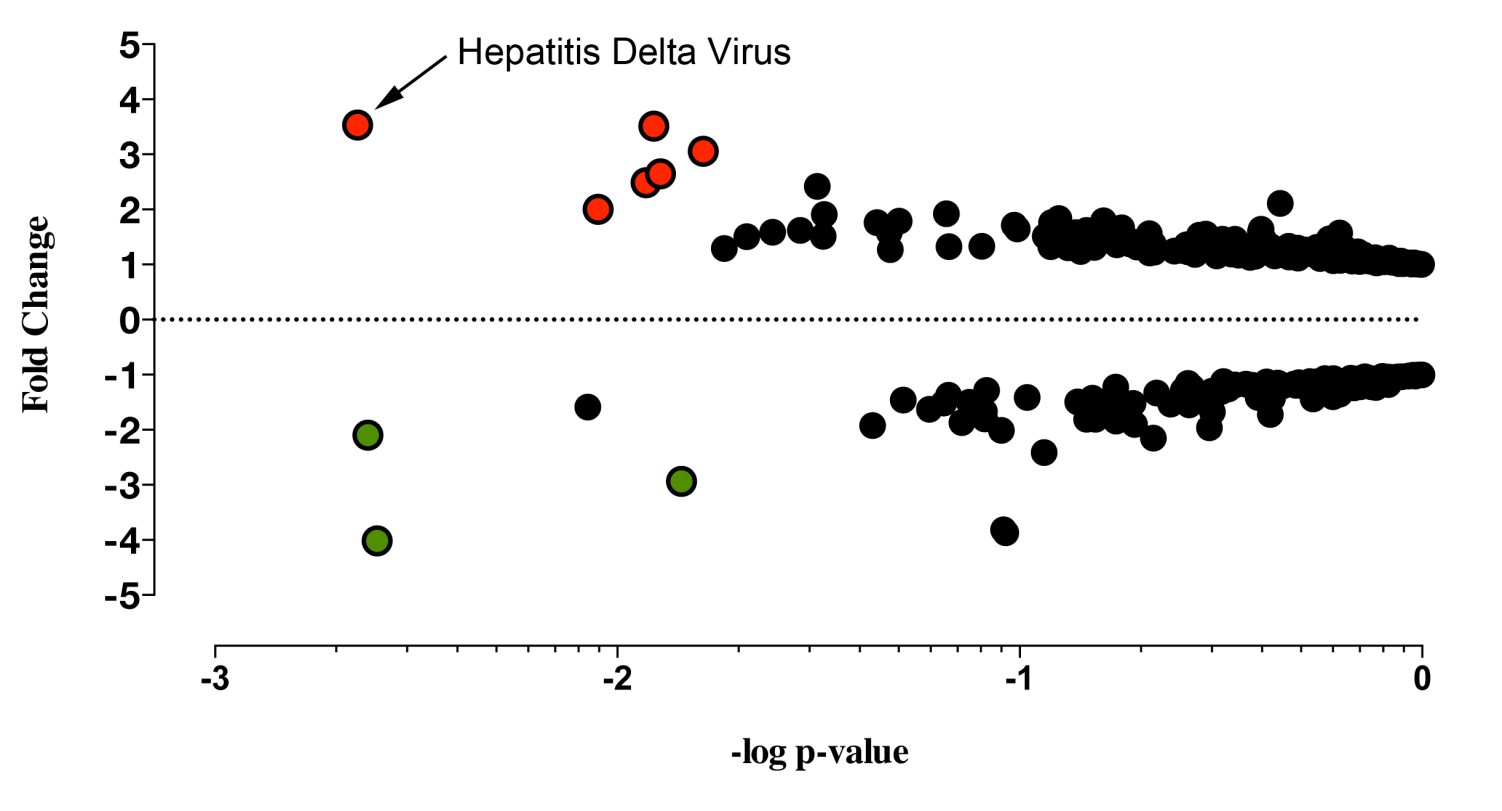
Collective analysis identified hepatitis delta virus in primary Sjögren's syndrome salivary gland tissue. Collective analysis comparing viral profiles of the pSS patient population to the healthy controls' viral profile identified 9 probes differentially expressed between the pSS population and the healthy controls. The graph denotes fold change and *P*-values (t-test) for probes above the background threshold. Red (positive fold change) and green (negative fold change) circles denote probes rendering a *P*-value of < 0.05 after Benjamini-Hochberg correction (*P*(Corr)< 0.05, t-test, Benjamini-Hochberg correction, n = 29). Black circles denote the remaining probes without a significant change between pSS patients and healthy controls after Benjamini-Hochberg correction (Supplemental Table 2).

The collective analysis of the viral transcripts differentially expressed between the pSS patient cohort and healthy controls identified 9 probes from 8 distinct viral families that were significantly altered in the pSS cohort. Six probes recognizing HDV, Herpesviridae, Retroviridae, Astroviridae, Adenoviridae, and Circoviridae viral families were significantly increased in pSS salivary gland tissue compared to healthy controls (Figure 1, Supplemental Table 2). Three probes recognizing Flaviviridae and Poxviridae viral families were significantly decreased in pSS compared to healthy controls. Through this collective analysis, HDV was identified as the most significant virus profile, presenting with the highest probe intensity and significant increase across the collective patient population compared to healthy controls.

**Figure 2.**
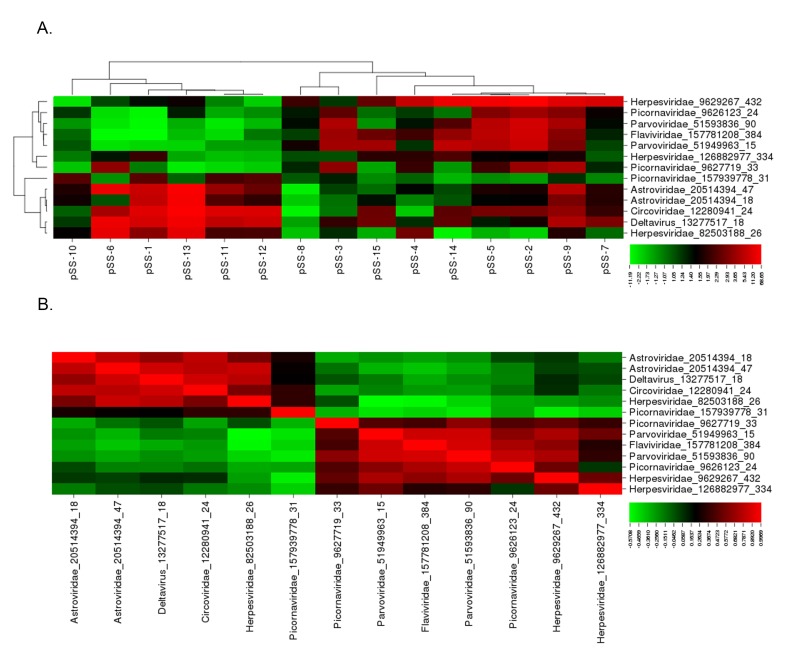
Subgroup analysis identified 2 distinct viral profiles present in the pSS patient population tested. (A) Cluster analysis of the 13 probes identified through subgroup analysis rendered 2 virus profiles. Virus profile I contained probes for deltavirus, Astroviridae, Circoviridae, and Herpesviridae. Virus profile II contained probes for Flaviviridae, Picornaviridae, Parvoviridae, and Herpesviridae. (B) Pairwise correlation of probes identified through subgroup analysis further defined the 2 divergent virus profiles.

The subgroup analysis of the viral transcripts differentially expressed within subpopulations in the pSS patient cohorts compared to the healthy controls confirmed the presence of two distinct viral profiles within the pSS patient population. Through the subgroup pSS patient analysis, 12 viral probes were significantly increased in subpopulations within the pSS patient cohort relative to the healthy controls (Figure 2A, Supplemental Table 3). Significant pairwise correlation analysis identified 2 distinct viral profiles within the pSS patient population (Figure 2B), Supplemental Table 4). Virus profile I contained probes recognizing the HDV, Astroviridae, Circoviridae, and Herpesviridae virus families. Virus profile II contained probes recognizing the Flaviviridae, Picornaviridae, Herpesviridae, and Parvoviridae virus families. Through this subgroup analysis, virus profile I, including HDV, presented the highest probe intensity and significant increase across the subgroup analysis.

Across both the collective and subgroup analyses, HDV was identified as the most significant virus signature in the pSS cohort present in the affected salivary gland tissue of Sjögren's syndrome patients, as defined by significantly elevated HDV probe intensity in the collective and individual pSS patients relative to healthy controls. Therefore, HDV was selected for further characterization and evaluation as a potential trigger in the development of a Sjögren's syndrome phenotype.

### Hepatitis Delta Virus Detected in pSS

**Figure 3.**
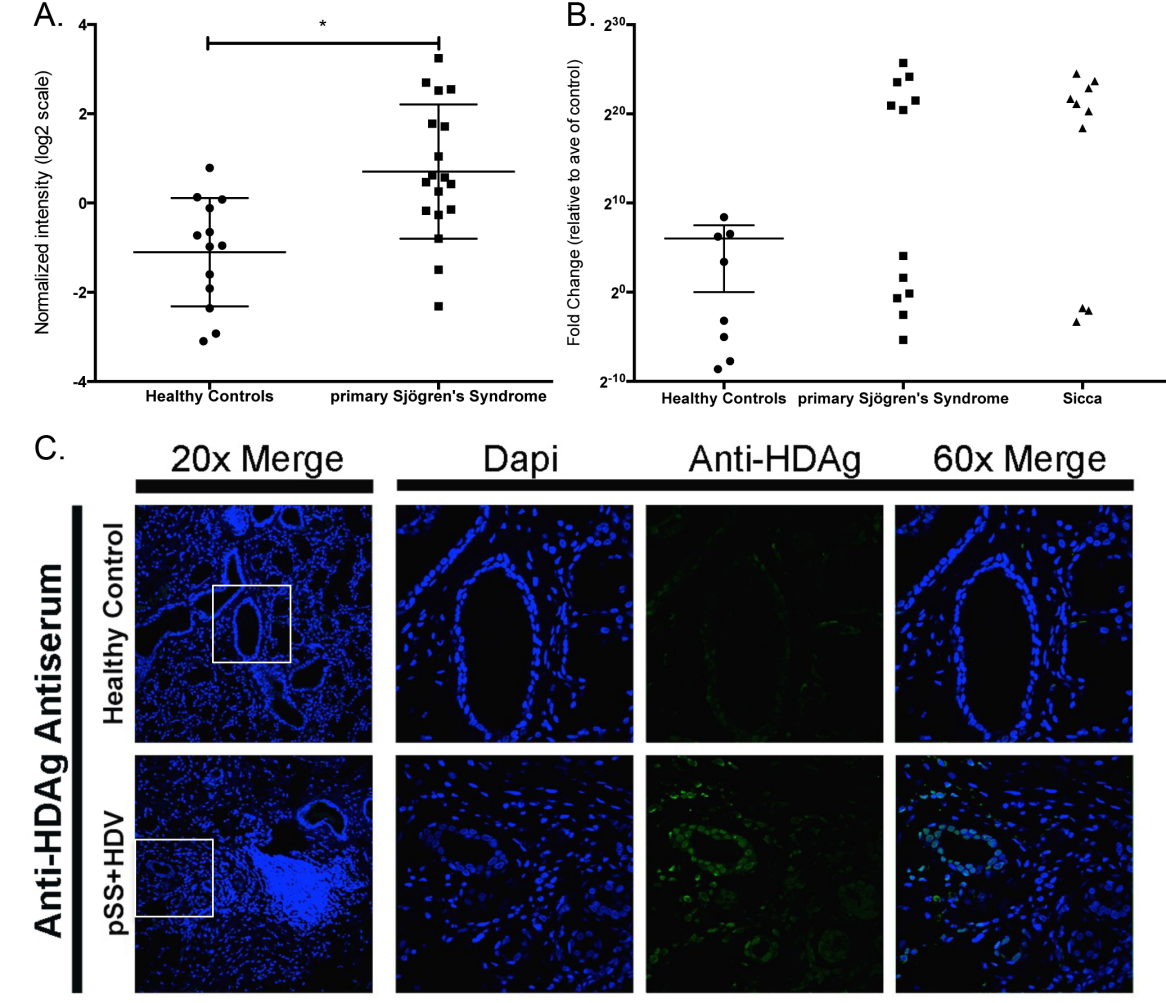
Viral microarray analysis identified HDV in 50% of the patients tested and was confirmed through detection of HDV sequence and antigen in salivary gland tissue. (A) Viral microarray analysis rendered a significant increase in HDV in pSS patients relative to healthy controls (*P* < 0.05, n = 26). (B) qPCR confirmed the presence of HDV sequence detected in 50% of pSS samples tested. Sicca patients were also positive for the presence of HDV sequence. Secondary confirmation was performed by an independent lab and detected HDV in 50% of samples tested (Supplemental Figure 1). (C) HDV antigens were detected in paraffin-embedded salivary gland tissue and rendered a nuclear staining pattern consistent with prior studies of HDV protein localization.

**Supplemental Figure 1.**
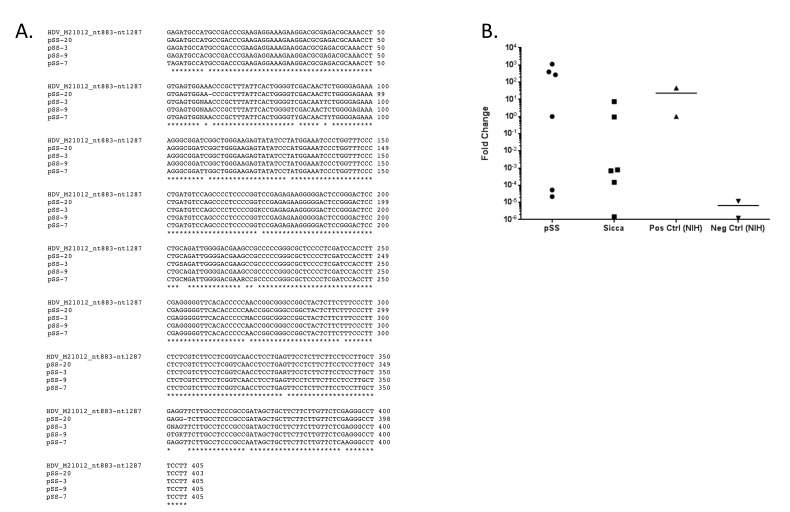
**HDV sequence was detected in salivary glands obtained from pSS and sicca patients.** A) Nested PCR was used to detect HDV genomic sequence in minor salivary gland tissue. Sequence homology (98.5% (97.3%-99.3%)) was observed within nested PCR. B) Nested qPCR retargeted to the region encompassing HDAg tran-script and genome was used to confirm the viral sequence present in a second pSS cohort and in a selection of patients experiencing sicca symptoms but did not meet full criteria for diagnosis. Two samples identified as positive and two samples identified as negative by qPCR performed on UK co-hort were independently processed and utilized as positive and negative controls in independent confirmation of HDV by nested qPCR in additional UK co-hort.

Microarray analysis identified the presence of hepatitis delta virus (HDV), which was significantly increased in over 50% of the pSS patients compared to healthy controls (Figure 3A-B). The HDV probe was homologous for the sequence from genotype 1, the genotype most prevalent in North America, Europe, and the Middle East [34]. Confirmation of HDV viral sequence was performed using nested RT-PCR [25] (Supplemental Figure 1A) using a new RT-qPCR assay targeted to the transcript region of the HDV genome (Figure 3B). To address the potential for contamination, the hepatitis delta virus sequence detected in minor salivary gland tissue was confirmed in a second pSS cohort in an independent lab with similar results (Supplemental Figure 1B). Hepatitis delta virus was also detected in a subset of sicca patients experiencing xerostomia and/or xerophthalmia symptoms but that did not meet the full criteria for pSS diagnosis at the time of tissue biopsy. No data were available at the time of analysis as to whether patients experiencing sicca symptoms had progressed to develop pSS. Hepatitis delta antigen was detected in HDV-positive pSS in comparison to salivary gland tissue from healthy controls using confocal immunofluorescence imaging (Figure 3C). Immunohistochemical detection of HDAg rendered a nuclear localization pattern in HDV-positive paraffin-embedded minor salivary gland tissue. These patterns are similar to those reported for HDV antigen expression in HDV infected tissues [28, 35].

### Absence of detectible past or present HBV infection

Primary Sjögren's syndrome patients positive for HDV in salivary gland tissue lacked evidence of a past or current hepatitis B virus (HBV) coinfection. Patient serum was tested for HBV surface antigen (HBsAg) and antibodies to HBV core (HBc) (Table 1). All of the patients tested for HBsAg were negative (Table 1). One healthy control and one pSS serum sample tested were positive for anti-HBc antibodies, but both of the samples positive for anti-HBc antibodies were negative for HDV sequence in salivary gland tissue (Supplemental Table 5). Luciferase Immunoprecipitation System (LIPS) assay offers a higher degree of sensitivity in the detection of antibodies [29]. The LIPS assay was also unable to detect anti-HBc antibodies in the serum of pSS patients that were positive for HDV in salivary gland tissue (Supplemental Table 5). These data are in line with reports of similar rates of HBc antibody detection between healthy controls and pSS [36]. Viral microarray contained over 100 probes recognizing hepadnaviridae virus family sequences but lacked significant differential expression between pSS and healthy controls or between HDV-positive pSS and healthy controls (Supplemental Table 6). Transaminase levels were within normal range for the pSS patient population tested and did not correlate with the level of HDV in salivary gland as detected by viral microarray (Supplemental Table 7). As well, the patients that tested positive for HDV by microarray lacked detectible antibodies to the HDV antigens (Table 1). In conclusion, pSS patients that tested positive for HDV in salivary gland tissue were negative for evidence of an active or past HBV infection or a systemic antibody production to HDV antigen.

**Table 1.** Clinical parameters of primary Sjögren's syndrome, primary Sjögren's syndrome-HOV_high_ or primary Sjögren's syndrome-HDV_low_ 10w relative to healthy control cohort.

	Healthy Controls	Primary Sjögren's Syndrome	Primary Sjögren's Syndrome	
			HDV_low_	HDV_high_
Age	42±9	50±13	54±12^#^	48±14
Sex (F/M)	8/7	8/7	3/4	5/3
Total Unstimulated Saliva Flow	4.77±5.15	1.22±1.48*	1.04±1.40	1.38±1.63
Total Stimulated Saliva Flow	18.28±11.27	9.14±6.49*	7.56±7.19^#^	10.53±5.94
Focus Score	0.36±0.63	1.87±1.25**	2.43±1.27^###^	1.37±1.06^#^
Schirmer's Test	19.2±12.07	5.5±4.9**	3.8±2.8^##^	7±6.0^#^
Anti-Nuclear Antibodies (% Positive)	0%	87%***	100%^###^	75%^##^
Anti-SSA /Ro Antibodies (% Positive)	0%	80%***	86%^###^	75%^###^
Anti-SSB/La Antibodies (% Positive)	0%	67%***	86%^###^	50%^#^
Rheumatoid Factor (% Positive)	17%	60%*	86%^#^	38%
lgG (Serum)	1146±269	1673±578*	1859±663^##^	1488±453
lgM (Serum)	137±85	104±37	95±37	113±38
C3 COMPLEMENT	124±22	112±29	105±23	118±35
C4 COMPLEMENT	26±10	21±9	21±8	21±10
WBC Count	5.76±1.44	5.09±1.44	5.39±1.79	4.79±1.02
Lymphocyte Count	30.7±6.2	36.0±8.7	34.4±8.8	37.6±9.0
Monocyte Count	7±3	9±1*	9±2	9±1
Hematocrit	42.1±2.9	41.6±1.4	41.7±2.4	41.4±4.6
Sed Rate	17±9	14±8	18±9	10±5
Hemoglobin	14.1±1.0	14.0±1.1	14.1±0.7	13.8±1.5
Platelets	281±45	251±76	248±89	255±66
Albumin	4.2±0.3	4.1±0.3	4.0±0.2	4.3±0.2†
Alkaline Phosphatase (ALP)	71±20	64±15	66±15	62±16
Alanine Transaminase (ALT)	24±11	27±11	24±7	30±14
Amylase	72±33	92±28	100±33	83±21
Aspartate Aminotransferase (AST)	24±6	31±23	26±6	37±32
Calcium	2.32±0.09	2.39±0.13	2.32±0.10	2.45±0.13
Chloride	104±3	104±1	103±1	104±1
Creatinine	0.80±0.18	0.84±0.16	0.83±0.18	0.84±0.15
Glucose	98±15	92±6	92±7	91±6
Magnesium	0.90±0.7	0.88±0.06	0.85±0.5	0.90±0.07

All values are mean± standard deviation unless otherwise stated. Statistically significant values in primary Sjögren's Syndrome versus healthy controls *P < 0.05, **P <0.005, ***P < 0.0005, n = 10-15. Statistically significant values in Primary Sjögren's Syndrome HDV_low_ versus Primary Sjögren's Syndrome HDV_high_ † *P* < 0.05, n = 6-8. Statistically significant values in Primary Sjögren's Syndrome HDV_high_ or Primary Sjögren's Syndrome HDV_low_ versus healthy controls #*P* < 0.05, ##*P* <0.005, ###*P* < 0.0005, n = 6-15. HDV_high_ is defined as samples possessing normalized microarray intensity values above 95% Confidence Interval based on healthy controls. HDV_low_ is defined as samples possessing normalized microarray intensity values within the 95% Confidence Interval based on healthy controls. Statistical analysis was evaluated by t-test or fisher's exact test for categorical data.

### Clinical Characteristics of HDV in pSS

The pSS patient population was divided into two groups, defined as pSS patients with elevated levels of HDV (positive/HDV_high,_ n = 8) and pSS patients with HDV levels similar to healthy controls (negative/HDV_low,_ n = 7), as determined by microarray analysis. Analyses were performed to identify significant changes in clinical parameters between the total pSS population and healthy controls, between HDV_high_ and HDV_low,_ between HDV_high_ pSS and healthy controls, and between HDV_low_ and healthy controls (Table 1).

Analysis of the total pSS patient cohort relative to healthy controls identified significant decreases in unstimulated saliva flow (*P* < 0.05), stimulated saliva flow (*P* < 0.05), and tear production (*P*< 0.005), and significant increases in focal lymphocytic infiltration (*P*< 0.005), and levels of total serum IgG and autoantibodies (anti-nuclear, anti-SSA/Ro, anti-SSB/La antibodies, and rheumatoid factor). There was a limited significant difference identified between the HDV_high_ and the HDV_low_ patient groups. Albumin was elevated in the HDV_high_ subgroup (4.3±0.2, *P* < 0.05) in comparison to the HDV_low_ cohort (4.0 ± 0.2). No significant difference was observed in albumin levels in the total pSS cohort in comparison to healthy controls.

Analysis of clinical parameters in HDV_high_ or HDV_low_ patients relative to healthy controls identified numerous significant differences in the multiple clinical features analyzed. While there was no significant difference in the age range of the healthy controls (42 ± 9) and total pSS population (50 ± 13, N.S.), pSS patients in the HDV_low_ subgroup were significantly older (54 ± 12, *P* <0.05) compared to healthy controls (42 ± 9), in contrast to HDV_high_ subgroup (48 ± 14). This study contained a higher percent of males (47%) than normally reported for Sjögren's syndrome patient populations (5-10%). No significant difference in sex ratio was observed between the HDV_low_ and HDV_high_ pSS groups. Total stimulated saliva flow was significantly decreased in the HDV_low_ pSS group (7.56 ± 7.19, *P* < 0.05) compared to healthy controls (18.28 ± 11.27). Focus score was significantly increased in both the HDV_low_ group (2.43 ± 1.27, *P* < 0.0001) and HDV_high_ group (1.37±1.06, *P* < 0.05). Tear production was significantly decreased in the HDV_low_ group (3.8 ± 2.8, *P* < 0.005) and HDV_high_ group (7.0 ± 6.0, *P* <0.05) relative to healthy controls (19.2 ± 12.07) as measured by Schirmer's test. Together, the HDV_high_ patient group displayed a less pronounced clinical presentation of Sjögren's syndrome symptoms and antibody profiles than the HDV_low_ patient group.

### HDV Antigens Induced a pSS-like Phenotype in Vivo

**Supplemental Figure 2.**
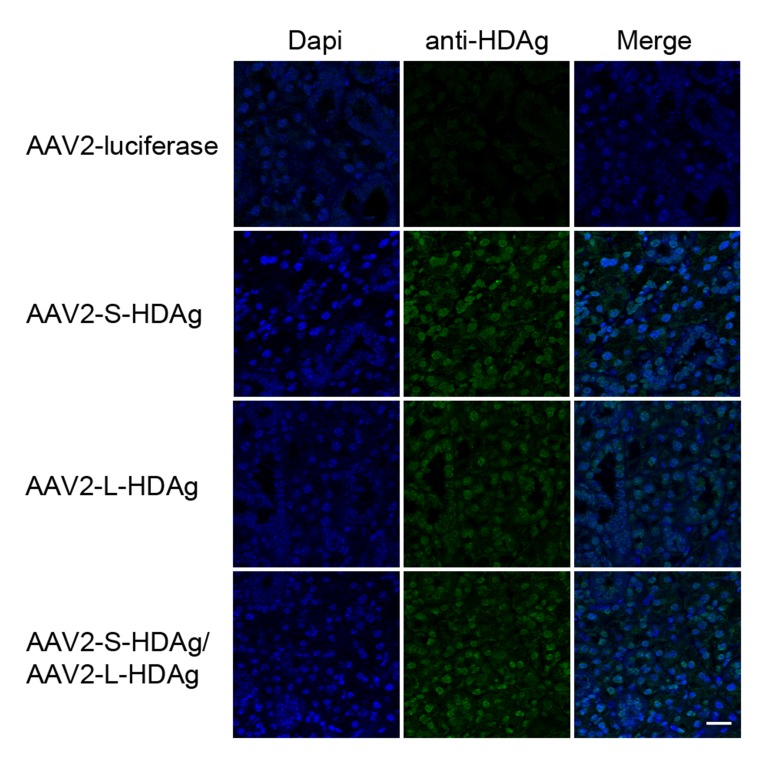
**Detection of HDAg in mouse salivary gland tissue in mice cannulated with AAV-HDAg.** Retrograde cannulation was used to delivery recombinant adeno-associated virus serotype 2 (rAAV-2) containing HDV genes to salivary gland tissue in female C57BL/6 mice. Salivary gland tissue was evaluated 4 months post cannulation for expression of HDAg (green), and dapi (blue) for nuclear localization. Scale bar 20μm

An animal model was developed to evaluate the potential of HDV antigens to initiate development of a Sjögren's syndrome phenotype *in vivo*. Hepatitis delta virus is a negative sense single strand RNA (ssRNA) virus that is classically thought to require a helper virus, hepatitis B virus (HBV), for packaging and transmission [37]. The ~1700nt circular HDV genome contains a single open reading frame and expresses two proteins, the small antigen (S-HDAg) and the large antigen (L-HDAg). The S-HDAg is responsible for initiation of RNA genome replication and is expressed early in the infection cycle. The L-HDAg is expressed in later stages of viral infection and plays a role in ribonucleoprotein (RNP) complex packaging. In the classic HBV:HDV coinfection model, the HDV genome, S-HDAg, and L-HDAg form the RNP complex and are packaged into the coat membrane of HBV. Due to the absence of HBV in the pSS viral profile, recombinant adeno-associated virus (rAAV) was used to deliver HDV genes to recapitulate the chronic HDV infection. Female C57BL/6 received rAAV containing S-HDAg, L-HDAg, or a combination of S-HDAg/L-HDAg expression cassettes through retrograde cannulation to salivary gland tissue. These three *in vivo* models depicted different stages of the HDV infection cycle. rAAV containing a luciferase expression cassette was utilized in the control group to account for rAAV-mediated responses. Immunohistochemical detection of HDAg rendered a nuclear localized pattern of protein expression as delivered by rAAV (Supplemental Figure 2). Mice were monitored for 4 months post-cannulation for development of autoantibodies, changes in stimulated saliva flow and focal lymphocytic infiltration in salivary gland tissue.

**Figure 4.**
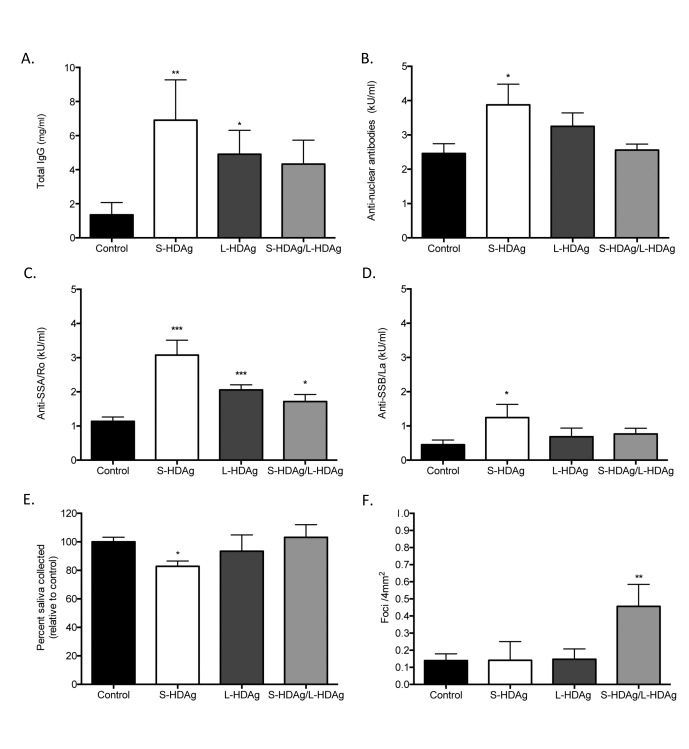
Expression of HDV antigens recapitulates a Sjögren's syndrome-like phenotype in an *in vivo* murine model. (A) Total IgG was significantly increased in mice expressing S-HDAg and L-HDAg, but not in mice that received combination S-HDAg/L-HDAg (t-test, **P < 0.01, *P < 0.05, n = 7-15). (B) Antinuclear antibodies (ANA) were elevated in mice expressing S-HDAg (t-test, *P < 0.05, n = 6-11). (C) Anti-SSA/ Ro60 antibodies were significantly elevated in mice expressing S-HDAg, L-HDAg, and the combination of S-HDAg/L-HDAg (t-test, ***P < 0.0005, *P < 0.05, n = 7-11). (D) An increase in Anti-SSB/La antibodies was observed in mice that expressed S-HDAg (t-test, *P < 0.05, n = 6-11). (E) Mice were cannulated with adeno-associated virus (AAV) containing expression cassettes for luciferase (control), S-HDAg, L-HDAg, or a combination of the two HDV antigens (S-HDAg/L-HDAg). Mice that expressed S-HDAg showed a significant decrease in pilocarpine stimulated saliva flow compared to controls (t-test, *P < 0.05, n = 6-14). (F) A significant increase in foci was noted in mice that expressed a combination of S-HDAg and L-HDAg (t-test, **P < 0.005, n = 6-14).

Expression of HDV antigens in mouse salivary gland tissue triggered the development of autoantibodies (Figure 4(A-D). Mice expressing S-HDAg, depicting an early HDV infection, showed a significant increase in total IgG, ANA, anti-SSA, and anti-SSB levels. Mice expressing L-HDAg showed a significant increase in total IgG and anti-SSA. Mice expressing a combination of S-HDAg and L-HDAg, depicting a later stage of HDV infection, showed a significant increase in anti-SSA. The strongest autoantibody profile was observed in mice expressing S-HDAg, representative of early stages of HDV infection.

Expression of S-HDAg in mouse salivary gland tissue resulted in a significant decrease in stimulated saliva flow (Figure 4E). Mice were monitored for pilocarpine-stimulated saliva flow at 4 months post cannulation. Mice expressing S-HDAg resulted in a statistically significant decrease in pilocarpine-stimulated saliva flow relative to controls. No change in pilocarpine-stimulated saliva production was noted in mice that expressed the L-HDAg or combined S-HDAg/L-HDAg expression. Together, our data suggest that the expression of S-HDAg, representative of early stages of HDV infection, possesses the capacity to impact stimulated saliva flow.

**Supplemental Figure 3.**
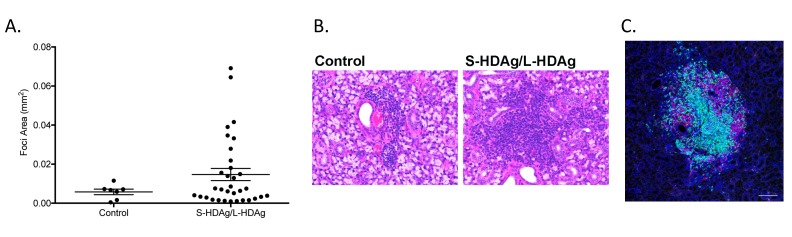
**Focal size of lymphocytic infiltrates were larger in mice expressing the combined S-HDAg and L-HDAg compared to foci size observed in mice expressing S-HDAg/L-HDAg in salivary gland tissue.** A) Area of foci in mice expression a combination of both the small and large HDV antigen revealed an increase in size a subset of the observed foci (n.s., n = 7-33). There was a significant increase in variance of size in the treated mice compared to the control mice (F-test, *P*=0.0008). B) Lymphocytic foci representative of the average size of foci present in control mice that expressed luciferase in salivary gland tissue (2.5E-3 mm2). Average size of foci in the mice expressing S-HDAg/L-HDAg is 13.1E-3mm2 in control mice. Images of lymphocytic foci representative of the average size of foci present. C) Ectopic lymphoid structures were evident in mice expressing combined S-HDAg/L-HDAg. Distribution of T-cell CD3 (green) and B-cell B220 (red) depict transition to organized lymphoid-like structures (bar 100um)

Expression of both S-HDAg and L-HDAg, depicting a later stage of HDV infection, resulted in a significant increase in focal lymphocytic infiltration in salivary gland tissue. Focal accumulation of lymphocytes within the affected salivary gland is a hallmark in pSS diagnosis. In contrast to the S-HDAg mediated reduction in saliva flow and strong autoantibody profile, mice that expressed combined S-HDAg/L-HDAg showed a significant increase in lymphocytic foci (Figure 4F). No significant increase in lymphocytic foci was noted with singular expression of S-HDAg or L-HDAg in the salivary glands compared to control mice (Figure 4F). The variance in the area of the foci in mice expressing the combination of S-HDAg and L-HDAg was observed to be increased compared to the area of foci in the control mice (Supplemental Figure 3A-B). Ectopic lymphoid structures were also evident in mice expressing combined S-HDAg/L-HDAg (Supplemental Figure 3C). Additionally, similar to the pSS patient cohort, mice expressing HDV antigens in salivary gland tissue did not develop detectible antibodies to HDV antigens (data not shown).

## DISCUSSION

This study identified the presence of HDV in a subset of Sjögren's syndrome patients. The detection of HDV by custom viral microarray was subsequently confirmed by detection of the HDV antigen in affected salivary gland tissue and detection of the HDV sequence in two pSS cohorts and by an independent research team. Animal models were developed to evaluate the potential for HDV antigens expressed in salivary gland tissue to trigger development of a Sjögren's syndrome-like phenotype. We have shown that the expression of HDV antigens in mouse salivary gland recapitulates the development of a complete pSS-like phenotype *in vivo*. Together, the identification of HDV in the affected salivary gland tissue of Sjögren's syndrome patients and the demonstrated ability of HDV antigens to trigger the pathogenesis of a pSS-like phenotype *in vivo* provide further support for a viral-mediated mechanism in the development of Sjögren's syndrome.

**Figure 5.**
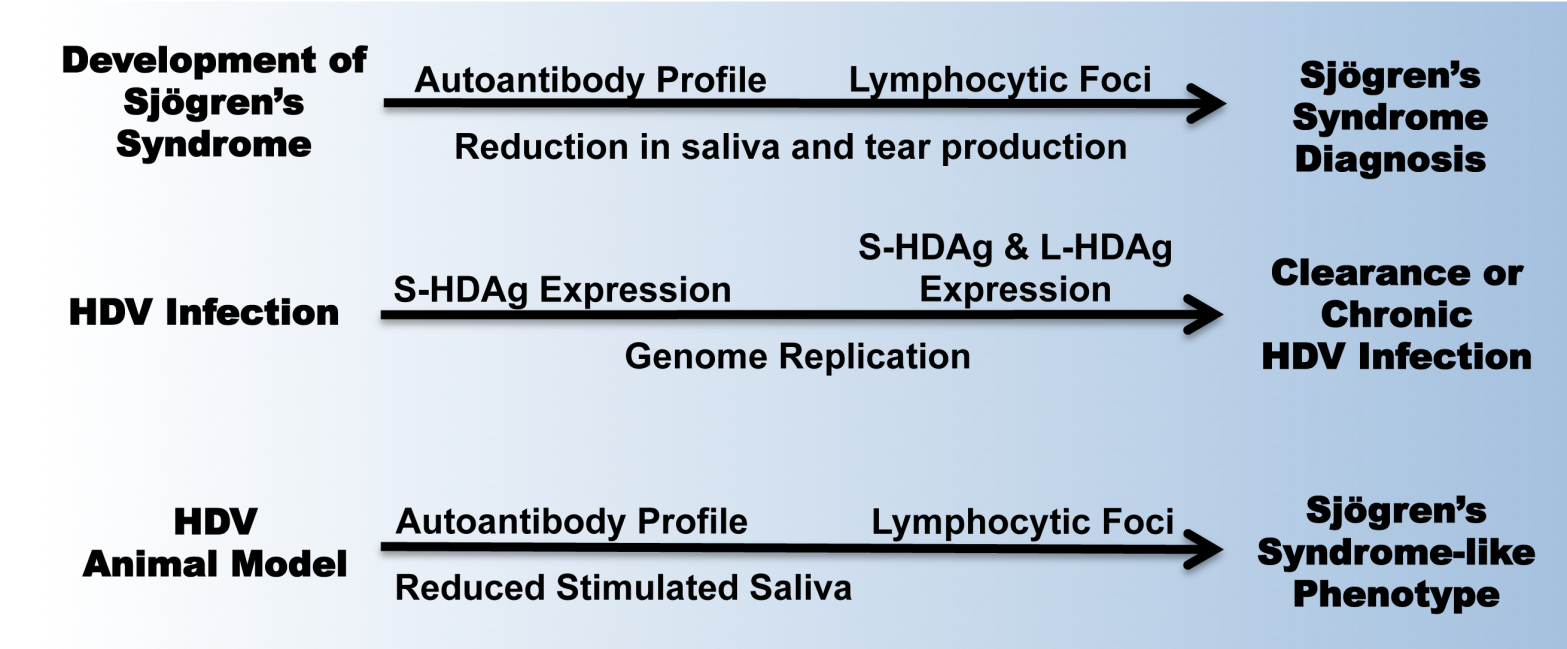
Progression of Sjögren's syndrome disease mirrors the development of HDV infection and HDV antigen-mediated induction of a Sjögren's syndrome-like phenotype *in vivo*.

The timeline for developing Sjögren's syndrome pathogenesis mirrors the HDV-mediated Sjögren's syndrome phenotype observed in our animal model and in the known HDV infection cycle (Figure 5). Sjögren's syndrome patients have reported subjective xerostomia and develop an autoantibody profile years prior to disease diagnosis [12–14]. Mirroring this behavior, the mice expressing S-HDAg, representing an early stage of HDV infection, presented the strongest autoantibody profile and significant reduction in stimulated saliva flow. In humans, lymphocytic infiltrates in the salivary gland tissue are evaluated and detected at later stages of pSS pathogenesis. In the mouse model, the combined expression of S-HDAg and L-HDAg, depicting a later stage HDV infection profile, resulted in a significant accumulation of lymphocytic foci *in vivo*. Together, the chronological development of Sjögren's syndrome echoes the progression of a chronic, low-grade HDV infection in the salivary gland as observed in our HDV animal model.

Chronic HDV infections have previously been associated with triggering a clear antiviral response and development of autoimmunity. Multiple studies have shown the capacity of HDV antigens to stimulate expression of or directly interact with multiple interferon-stimulated genes and genes associated with pSS pathogenesis, including SSB/La protein [38–41]. Prior studies have associated HDV infections with the development of autoantibodies, including ANA, liver-kidney microsomal antibodies (LKM), and smooth muscle antibodies (SMA), among others [42–44]. Our data, in connection with prior reports of HDV-mediated autoantibody development, further support the potential of HDV to trigger development of pSS-associated autoimmunity. The type of autoantibody profile developed in the presence of HDV may be dependent on the tissues and cellular localization of active HDV persistence.

Detection of HDV in the salivary gland tissue of Sjögren's syndrome patients in the absence of HBV points to a unique tissue tropism and/or mechanism of persistence not previously identified. Hepatitis delta virus can readily replicate its genome and antigens using host cellular mechanisms, enabling persistence in the absence of an active helper virus infection, and has been shown to persist in tissue culture at low levels for at least a year in the absence of a helper virus [27, 37, 38]. Therefore, once inside the cell, HDV is able to establish a chronic presence. In our hands, evidence of a past or current HBV infection has not been identified in affected salivary gland tissue in pSS patients that are HDV positive. This is in line with prior studies evaluating the incidence of HBV infection in pSS studies that have found similar or lower HBV infection rates in pSS patients [36, 45, 46]. The question remains as to how HDV gains entry into the salivary glands of pSS patients in the absence of a detectible current or past HBV infection. Prior studies have shown cell-mediated immune response to low-level viral infections and/or exposures in the absence of antibody production [47]. Further studies are needed to evaluate the potential of cell-mediated immune response to low level HDV and HBV antigens in the absence of a detectible humoral immune response in both the pSS patient population and HDV animal models.

Further analysis is needed to identify primary routes of infection and viable antiviral therapeutics. Prior studies have shown the sequence homology of genotype 1 HDV infections associated with HDV:HBV coinfection ranges between 91.2% and 91.7% [48, 49]. In our study, a higher percent of sequence homology of 98.5% (between 97.3%-99.3%) was observed in pSS patients. This high degree of sequence homology between patient samples may be a factor of the low level rate of genome replication or a stable primary reservoir. Multiple therapies are being evaluated for treatment of chronic HDV infection associated with HBV coinfection, including lonafarnib, tipifarnib, and FTI-277 [50, 51]. The primary mechanism of these drugs inhibits farnesyl transferase activity, which is required for the post-translational modification of the L-HDAg facilitating interaction with HBsAg and packaging in the HBV virion. While these drugs have shown success in treating HDV in relation to HDV:HBV coinfection, it is unclear at this stage whether these drugs would have a similar effect in the chronic HDV present in salivary gland tissues of Sjögren's syndrome patients and in the absence of an active HBV infection. Ongoing studies are focused on how HDV gains entry into salivary gland tissue in the absence of a detectible HBV infection and the underlying mechanism(s) of HDV-mediated pSS disease pathogenesis.

Multiple viral infections have been associated with the development of sialadenitis and sicca symptoms similar to that in pSS patients. As primary examples, hepatitis C virus (HCV) and human immunodeficiency virus (HIV) infections are noted to trigger sialadenitis, sicca symptoms, and distinct autoantibody profiles [52–54]. Identifying the association between these viral infections and Sjögren's syndrome symptoms has refocused the therapeutic approach to treating the underlying viral infection. HIV and HCV are now exclusion criteria in the diagnosis of Sjögren's syndrome [11]. The identification of HDV in pSS patients and the recapitulation of disease phenotype *in vivo* follows a similar path of study previously reported for HCV in pSS populations and *in vivo* [52, 55]. Therefore, we postulate that HDV may be another viral infection resulting in the development of a Sjögren's syndrome-like phenotype, and further analysis is warranted to evaluate the diagnosis of a chronic HDV infection as an exclusion criterion for the diagnosis of Sjögren's syndrome. Identifying the Sjögren's syndrome patient population that is HDV positive will refocus the therapeutic approach from generalized immune suppression to targeted antiviral therapies.

Prior studies that have introduced the expression of viral proteins, including HCV and HTLV-1 proteins, or endogenous proteins associated with pSS, including bone morphogenic protein 6 (BMP-6), have shown incomplete primary Sjögren's syndrome-like phenotypes [24, 55, 56]. Most notably, these prior studies have lacked the production of pSS-associated autoantibodies to SSA/ Ro or SSB/La proteins. Our study identified the increased presence of HDV in the salivary gland tissue of pSS patients and targeted expression of HDV antigens in murine salivary glands resulted in the development of a complete Sjögren's syndrome phenotype. Together, these data suggest that the simple expression of a viral or endogenous protein or general viral infections in the salivary gland are not sufficient to elicit a complete pSS-like disease phenotype and that the pSS disease pathogenesis may be a targeted response to a specific viral exposure.

Analysis of clinical features based on the level of HDV within affected salivary gland tissue revealed a less pronounced disease symptomology in the HDV_high_ pSS patients. The detection of HDV in patients experiencing sicca symptoms but not meeting full criteria for pSS diagnosis may provide an early window for diagnosis and intervention prior to progression of pSS-associated pathology. Pertovaara *et al*. reported that 36% of patients experiencing sicca symptoms progress to development of pSS [57]. Our current hypothesis is that HDV-positive sicca patients may either be at early stages of the disease progression or are missing a cellular or genetic susceptibility factor required for progression of disease pathology. Further studies are needed to clarify the clinical differences between HDV_high_ and HDV_low_ pSS patients and to evaluate disease progression or resolution in HDV-positive sicca patients to help define the susceptibility factors supporting or restricting chronic persistence of HDV in salivary gland tissue.

Primary Sjögren's syndrome is not considered to be an infectious or contagious disease. Therefore multiple factors, including genetic susceptibility, favorable cellular and/or tissue environment, and/or exposure to pathogen(s), may be required to promote a viral-mediated development of pSS pathology. Further large-scale studies are needed to define the incidence of HDV in global pSS patient cohorts and the underlying genetic susceptibility factors impacting HDV-mediated disease pathogenesis.

## SUPPLEMENTARY MATERIALS

**Supplemental Table 1.** Summary of primary Sjögren's syndrome (pSS) and Sicca patient samples uti-lized in analysis of viral profiles present in Sjögren's syndrome patient cohorts at NIH and in UK.

NIH Patient ID	Sex	Schirmer's Test	Unstimulated Saliva Flow	Focus Score (≥ 1)	Autoantibody	Diagnosis
pSS-1	Male	Negative	Low Flow	Negative	Positive	pSS
pSS-2	Female	Positive	Low Flow	Positive	Positive	pSS
pSS-3	Male	Positive	Low Flow	Positive	Positive	pSS
pSS-4	Female	Negative	Low Flow	Positive	Positive	pSS
pSS-6	Female	Positive	Low Flow	Positive	Positive	pSS
pSS-7	Male	Negative	Low Flow	Positive	Positive	pSS
pSS-9	Female	Positive	Low Flow	Negative	Positive	pSS
pSS-10	Female	Positive	Low Flow	Positive	Positive	pSS
pSS-11	Male	Positive	Normal Flow	Positive	Positive	pSS
pSS-12	Female	Positive	Low Flow	Positive	Negative	pSS
pSS-13	Female	Positive	Low Flow	Positive	Negative	pSS
pSS-14	Female	Negative	Normal Flow	Positive	Positive	pSS
pSS-15	Male	Negative	Low Flow	Positive	Positive	pSS
pSS-19	Male	Positive	Normal Flow	Positive	Positive	pSS
pSS-20	Male	Positive	Normal Flow	Positive	Positive	pSS

UK Patient ID	Sex	Schirmer's Test	Unstimulated Saliva Flow	Focus Score (≥ 1)	Autoantibody	Diagnosis
UK-1	Female	Positive	Normal Flow	Positive	Positive	pSS
UK-2	Male	Positive	Normal Flow	Positive	Positive	pSS
UK-3	Female	Positive	Low Flow	Positive	Positive	pSS
UK-4	Female	Positive	Low Flow	Positive	Positive	pSS
UK-5	Female	Positive	Low Flow	Positive	Positive	pSS
UK-6	Female	Negative	Normal Flow	Positive	Positive	pSS
UK-7	Female	Positive	Low Flow	Positive	Positive	pSS
UK-8	Female	Positive	Low Flow	Positive	Positive	pSS
UK-9	Female	Positive	Low Flow	Positive	Positive	pSS
UK-10	Female	Positive	Normal Flow	Positive	Positive	pSS
UK-11	Female	Positive	Normal Flow	Positive	Positive	pSS
UK-12	Male	Positive	Normal Flow	Positive	Positive	pSS
UK-13	Female	Positive	Low Flow	Negative	Negative	Sicca
UK-14	Female	Negative	Normal Flow	Negative	Negative	Sicca
UK-15	Female	Negative	Normal Flow	Negative	Negative	Sicca
UK-16	Female	Negative	Normal Flow	Negative	Negative	Sicca
UK-17	Female	Positive	Low Flow	Negative	Negative	Sicca
UK-18	Male	Positive	Low Flow	Negative	Negative	Sicca
UK-19	Female	Positive	Low Flow	Negative	Negative	Sicca
UK-20	Female	Negative	Normal Flow	Negative	Negative	Sicca
UK-21	Female	Positive	Normal Flow	Negative	Negative	Sicca
UK-22	Female	Positive	Normal Flow	Negative	Negative	Sicca

All pSS patients met diagnosis criteria based on the American European consensus group (AECG).

**Supplemental Table 2.** Viral microarray analysis identified 9 viral probes with significantly different intensity between the collective pSS patient popu-lation compared to healthy controls.

Family Name	Probe Name	Sequence	P-value	P (Corr)	FC
Deltavirus	Deltavirus_13277517_18	GGTCAACCTCCTAAGTTCCTCTTCCTCCTCCTTGCTGAGGTTCTTTCCCCCCGCCGATAG	0.004	0.022	3.25
Herpesviridae	Herpesviridae_9629267_432	GCCCTGGGCCCCGAGGCCATCCAGGCGCGGCTGGAGGACGTGCGGATCCAGGCCCGCCGG	0.012	0.033	3.47
Astroviridae	Astroviridae_20514394_47	CAACCTCAACCTCAACCTCCTGCTCCTATTGAAGAAATACTTCTGCCTTTAGCAGAACTG	0.014	0.033	2.60
Adenoviridae	Adenoviridae_34303903_34	TTCACTAAGTTTGCCTCCAGCTCTACAAAAAATTTTAATTGAAATTTTTATTGGAAAATC	0.014	0.033	2.48
Retroviridae	Retroviridae_9629902_75	CATTGCAAGTTATTGCGATCATCTCTCTCCTCCTCGTTGGGGGGGCCAGTCAACCAGCTA	0.009	0.033	2.05
Circoviridae	Circoviridae_12280941_24	TGCCAACTTTGTAACCCCCTCCACCAACTTGGCCTATGACCCCTATATTAACTACTCCTC	0.023	0.044	2.86
Flaviviridae	Flaviviridae_157781216_244	TATACCAATGCTGTAACCTTGAACCGGAGGCCAGGAAAGTGATCTCCTCCCTCACGGAGC	0.003	0.022	-2.10
Poxviridae	Poxviridae_96980809_55	GCTTCCGCAAGGCGGTCGAGCGGCAGCTGGAGGAGGAGCAGGAGGTGAAGCTGAAGAGCC	0.003	0.022	-4.00
Poxviridae	Poxviridae_96980809_110	TCGAGCGGCAGCTGGAGGAGGAGCAGGAGGTGAAGCTGAAGAGCCTCGCGCGGCGCGAGT	0.016	0.033	-2.93

Six probes were increased in pSS compared to healthy controls and 3 probes were significantly decreased in pSS compared to healthy controls. (FC = Fold Change, *P*-value denotes t-test, *P*(Corr) denotes *P* < 0.05, t-test, Benjamini-Hochberg correction n = 14-15)

**Supplemental Table 3.** Analysis of viral microarray data for subgroups within the Sjögren's syndrome patient population identified 12 viral probes sig-nificantly increased in at least 25% of the patient co-hort.

Viral Family Name	Probe Name	Sequence
Astroviridae	Astroviridae_20514394_18	CCAACGACAGGAGCTCAACCTCAACCTCAACCTCCTGCTCCTATTGAAGAAATACTTCTG
Astroviridae	Astroviridae_20514394_47	CAACCTCAACCTCAACCTCCTGCTCCTATTGAAGAAATACTTCTGCCTTTAGCAGAACTG
Parvoviridae	Parvoviridae_51949963_15	ACACCATCTGGCTGTTTGGACCCGCCACCACCGGCAAGACCAACATTGCGGAAGCCATCG
Adenoviridae	Adenoviridae_46852402_27	ACCGCCTCCACCCTGGAGGCCATGCTGCGCAACGACACCAACAACCAAACCTTTATTGAC
Circoviridae	Circoviridae_12280941_24	TGCCAACTTTGTAACCCCCTCCACCAACTTGGCCTATGACCCCTATATTAACTACTCCTC
Herpesviridae	Herpesviridae_126882977_334	CAGCCCTCTCAGCCCAAAGCCATTACTAAAGTCCCGTTGTATGTACAAGCGCAAGAGGAA
Picornaviridae	Picornaviridae_9626123_24	ATAGCCTACACCCCGCCTGGTGCGGGAAAGCCCACCACCCGGGACCAAGCTATGCAGGCT
Picornaviridae	Picornaviridae_157939778_31	TCGAGAGCAACCCTGGCCCTCTGTATGTTTGTTCCCAACCAGGTAAGTGCGATCCATTTC
Herpesviridae	Herpesviridae_9629267_432	GCCCTGGGCCCCGAGGCCATCCAGGCGCGGCTGGAGGACGTGCGGATCCAGGCCCGCCGG
Deltavirus	Deltavirus_13277517_18	GGTCAACCTCCTAAGTTCCTCTTCCTCCTCCTTGCTGAGGTTCTTTCCCCCCGCCGATAG
Flaviviridae	Flaviviridae_157781208_384	TTTATCACAGCATGTCTCATGCCCGACCCCGCTATTTACTCCTGTGCCTACTCATACTTA
Parvoviridae	Parvoviridae_51593836_90	TCAACAAGAGGAACACCGTCTGGCTCTACGGACCCGCCACGACCGGCAAGACCAACATCG

Subgroup analysis was performed through identifying normalized probe intensity of each individual patient that was greater than the average +2 standard deviations. Probes selected for further analysis were found in > 25% of the pSS samples tested.

**Supplemental Table 4.** Significant pairwise correlations identified between probes significantly increased in > 25% of the pSS patient population compared to healthy controls.

Variable	by Variable	Correlation	Count	Lower 95%	Upper 95%	Signif Prob
Deltavirus_13277517_18	Astroviridae_20514394_47	0.9268	15	0.7892	0.9758	< 0.0001
Flaviviridae_157781208_384	Parvoviridae_51949963_15	0.9121	15	0.7506	0.9708	< 0.0001
Parvoviridae_51593836_90	Picornaviridae_9626123_24	0.9097	15	0.7443	0.97	< 0.0001
Parvoviridae_51593836_90	Flaviviridae_157781208_384	0.9056	15	0.7336	0.9685	< 0.0001
Flaviviridae_157781208_384	Picornaviridae_9626123_24	0.8666	15	0.6373	0.9549	< 0.0001
Circoviridae_12280941_24	Astroviridae_20514394_18	0.8614	15	0.6248	0.9531	< 0.0001
Parvoviridae_51593836_90	Parvoviridae_51949963_15	0.8607	15	0.6231	0.9528	< 0.0001
Circoviridae_12280941_24	Astroviridae_20514394_47	0.8038	15	0.4956	0.9322	0.0003
Deltavirus_13277517_18	Circoviridae_12280941_24	0.8094	15	0.5076	0.9343	0.0003
Astroviridae_20514394_47	Astroviridae_20514394_18	0.7947	15	0.4764	0.9288	0.0004
Parvoviridae_51593836_90	Herpesviridae_9629267_432	0.7615	15	0.4087	0.9163	0.001
Herpesviridae_9629267_432	Parvoviridae_51949963_15	0.7122	15	0.3148	0.8972	0.0029
Herpesviridae_9629267_432	Picornaviridae_157939778_31	-0.7074	15	-0.8952	-0.306	0.0032
Picornaviridae_9626123_24	Parvoviridae_51949963_15	0.7059	15	0.3034	0.8947	0.0033
Deltavirus_13277517_18	Parvoviridae_51949963_15	-0.6949	15	-0.8903	-0.2837	0.004
Deltavirus_13277517_18	Astroviridae_20514394_18	0.6884	15	0.2721	0.8877	0.0045
Flaviviridae_157781208_384	Deltavirus_13277517_18	-0.6727	15	-0.8813	-0.2447	0.006
Flaviviridae_157781208_384	Herpesviridae_9629267_432	0.6656	15	0.2326	0.8784	0.0068
Parvoviridae_51949963_15	Astroviridae_20514394_47	-0.6512	15	-0.8724	-0.2084	0.0086
Parvoviridae_51593836_90	Deltavirus_13277517_18	-0.6162	15	-0.8577	-0.1519	0.0144
Herpesviridae_9629267_432	Picornaviridae_9626123_24	0.5904	15	0.112	0.8466	0.0205
Flaviviridae_157781208_384	Astroviridae_20514394_47	-0.5871	15	-0.8452	-0.107	0.0214
Herpesviridae_9629267_432	Herpesviridae_126882977_334	0.5414	15	0.0403	0.8249	0.0371
Picornaviridae_157939778_31	Parvoviridae_51949963_15	-0.5293	15	-0.8194	-0.0233	0.0425
Flaviviridae_157781208_384	Picornaviridae_157939778_31	-0.5287	15	-0.8191	-0.0225	0.0428
Deltavirus_13277517_18	Picornaviridae_9626123_24	-0.5253	15	-0.8176	-0.0179	0.0443

Significant pairwise correlations between viral probes revealed two distinct viral profiles present within the pSS patient population. (PCC, n = 15, *P*>0.05. Table is ranked on statistical significance of correlation.

**Supplemental Table 5.** Evidence of past or current hepatitis B virus (HBV) infection or antibodies to HDV not detected in HDV positive pSS patients.

Detection by Elisa	Healthy Controls (%)	primary Sjögren's syndrome (%)
HBsAg	0/11 (0%)	0/13 (0%)
Anti-HBc Elisa	1/8 (12.5%)	1/13 (7.7%)#
Anti-HBc LIPS	2/7 (28.6%)	1/12 (8.3%)#
Anti-HDAg Elisa	0/8 (0%)	0/21 (0%)
Anti-HDAg LIPS	0/7(0%)	0/10 (0%)

Antibodies to HDAg and Hepatitis B virus core (HBc) and HBV surface antigen (HBsAg) were quantified in serum by ELISA and the LIPS assay.

# The pSS patient positive for anti-HBc was negative for HDV within the salivary gland.

**Supplemental Table 6.** Analysis of Hepadnaviridae probes on viral microarray did not find evidence of HBV differentially expressed in pSS or in pSS that possessed HDV in salivary gland tissue relative to healthy controls.

		pSS versus Healthy Controls		HDV+pSS versus Healthy Controls		HDV-pSS versus Healthy Controls	
Family Name	Probe Name	P (Corr)	FC	P (Corr)	FC	P (Corr)	FC
Hepadnaviridae	Hepadnaviridae_9626719_104	0.92	1.05	0.80	1.33	0.84	-1.34
Hepadnaviridae	Hepadnaviridae_48696569_109	0.88	-1.25	0.80	-1.69	0.85	1.26
Hepadnaviridae	Hepadnaviridae_48696604_116	0.84	-1.44	0.80	-1.52	0.85	-1.34
Hepadnaviridae	Hepadnaviridae_22256030_12	0.72	-1.23	0.80	-1.21	0.84	-1.26
Hepadnaviridae	Hepadnaviridae_9630370_13	0.51	-1.91	0.62	-2.02	0.84	-1.75
Hepadnaviridae	Hepadnaviridae_21326584_14	0.88	-1.15	0.91	1.08	0.84	-1.59
Hepadnaviridae	Hepadnaviridae_9630370_14	0.77	1.59	0.88	1.27	0.78	2.21
Hepadnaviridae	Hepadnaviridae_9630370_15	0.88	1.20	0.80	1.42	0.91	-1.08
Hepadnaviridae	Hepadnaviridae_21326584_16	0.72	-2.05	0.80	-2.60	0.85	-1.43
Hepadnaviridae	Hepadnaviridae_21326584_16	0.72	1.38	0.80	1.37	0.84	1.38
Hepadnaviridae	Hepadnaviridae_9628827_17	0.77	1.13	0.92	-1.02	0.54	1.38
Hepadnaviridae	Hepadnaviridae_48696604_18	0.91	-1.23	0.80	-1.53	0.91	1.13
Hepadnaviridae	Hepadnaviridae_9626719_20	0.72	1.50	0.80	1.63	0.85	1.31
Hepadnaviridae	Hepadnaviridae_9630370_20	0.72	-1.15	0.80	-1.06	0.78	-1.31
Hepadnaviridae	Hepadnaviridae_21326584_21	0.77	-1.77	0.80	-2.07	0.85	-1.39
Hepadnaviridae	Hepadnaviridae_22256030_22	0.76	1.29	0.80	1.45	0.86	1.08
Hepadnaviridae	Hepadnaviridae_9628827_22	0.45	1.52	0.27	1.74	0.78	1.24
Hepadnaviridae	Hepadnaviridae_9630370_23	0.91	-1.16	0.92	1.06	0.85	-1.60
Hepadnaviridae	Hepadnaviridae_22256030_29	0.51	1.36	0.55	1.51	0.84	1.15
Hepadnaviridae	Hepadnaviridae_9628827_29	0.45	1.52	0.27	1.79	0.84	1.18
Hepadnaviridae	Hepadnaviridae_9628827_3	0.72	1.34	0.80	1.23	0.53	1.53
Hepadnaviridae	Hepadnaviridae_9630370_3	0.98	1.01	0.91	1.11	0.85	-1.15
Hepadnaviridae	Hepadnaviridae_9628827_8	0.91	1.07	0.80	-1.36	0.54	1.87
Hepadnaviridae	Hepadnaviridae_9630370_8	0.92	1.06	0.91	1.11	0.99	-1.01
Hepadnaviridae	Hepadnaviridae_21326584_9	0.72	1.51	0.80	1.84	0.85	1.12
Hepadnaviridae	Hepadnaviridae_22256030_9	0.77	1.25	0.88	-1.12	0.48	2.06
Hepadnaviridae	Hepadnaviridae_9630370_9	0.88	1.22	0.80	1.54	0.85	-1.17

Evaluation of HBV transcripts as detected by the viral microarray showed no significant difference in HBV transcripts levels between pSS patient samples and healthy controls showed. Evaluation of just the 9 samples that presented with HDV probe intensity above the 99%CI of HDV probe intensity of the healthy controls did not reveal a differential expression in pSS with HDV in salivary gland tissue compared to healthy controls. No significant difference in HBV probe intensity was noted between the HDV-negative pSS patients and healthy controls. (n = 14-15, t-test, Benjamini-Hochberg correction (P Corr), was utilized to calculate statistical significance. FC = fold change)

**Supplemental Table 7.** Correlations between clinical parameters and HDV levels in minor salivary gland biopsy.

Variable	By Variable	Correlation	Signif Prob
Anti-ANA Antibody	HDV	0.4475	0.0420*
Anti-SSA/Ro Antibody	HDV	0.5082	0.0187*
Anti-SSB/La ntibody	HDV	-0.0398	0.8639
Albumin	HDV	-0.0624	0.7937
Aspartate Aminotransferase (AST)	HDV	-0.1075	0.6519
Alanine Aminotransferase (ALT)	HDV	-0.2411	0.3058
Alkaline Phosphatase (ALP)	HDV	-0.277	0.2372

Select autoantibodies in serum significantly correlated with levels of HDV detected in minor salivary glands of healthy volunteers compared to patients diagnosed with primary Sjögren's syndrome. Levels of ANA and Anti-SSA/Ro antibody significantly correlated with levels of HDV as determined by microarray detection. Liver function tests, including measurement of serum aspartate aminotransferase (AST) and alanine aminotransferase (ALT) were all within normal range (data not shown) and did not significantly correlate with levels of HDV detected in salivary gland tissue. (PCC,*P<0.05, n = 21-23).
